# Enzymatically Crosslinked In Situ Synthesized Silk/Gelatin/Calcium Phosphate Hydrogels for Drug Delivery

**DOI:** 10.3390/ma14237191

**Published:** 2021-11-25

**Authors:** Andra Grava, Karina Egle, Arita Dubnika

**Affiliations:** 1 Rudolfs Cimdins Riga Biomaterials Innovations and Development Centre of RTU, Faculty of Materials Science and Applied Chemistry, Institute of General Chemical Engineering, Riga Technical University, Pulka 3/3, LV-1007 Riga, Latvia; andra.grava@rtu.lv (A.G.); karina.egle@rtu.lv (K.E.); 2 Baltic Biomaterials Centre of Excellence, Headquarters at Riga Technical University, LV-1658 Riga, Latvia

**Keywords:** silk fibroin, calcium phosphates, composite materials, hydrogels, drug delivery, 3D visualization, cell viability

## Abstract

Our research focuses on combining the valuable properties of silk fibroin (SF) and calcium phosphate (CaP). SF is a natural protein with an easily modifiable structure; CaP is a mineral found in the human body. Most of the new age biocomposites lack interaction between organic/inorganic phase, thus SF/CaP composite could not only mimic the natural bone, but could also be used to make drug delivery systems as well, which can ensure both healing and regeneration. CaP was synthesized in situ in SF at different pH values, and then crosslinked with gelatin (G), horseradish peroxide (HRP), and hydrogen peroxide (H_2_O_2_). In addition, dexamethasone phosphate (DEX) was incorporated in the hydrogel and drug delivery kinetics was studied. Hydrogel made at pH 10.0 was found to have the highest gel fraction 110.24%, swelling degree 956.32%, and sustained drug delivery for 72 h. The highest cell viability was observed for the hydrogel, which contained brushite (pH 6)—512.43%.

## 1. Introduction

Bone and cartilage damage is the main cause of osteoarthritis, but other factors, such as joint injures, obesity, bone deformities, multiple stress on joints, and metabolic diseases, increase it [1]. Osteoarthritis is the eighth leading health condition associated with disability [2]. Muscle mass tends to decrease with age, which affects musculoskeletal functions, for example, lower gait speed and weaker hand grip [2]. Thanks to tissue engineering, defective areas can be mended and cured. The new-age materials are no longer one-phase materials as they cannot provide all the necessary properties. This is where composite materials come in.

Composites are made by combining two or more materials to enhance their individual properties. Composite materials can be divided into many groups, for example, by scale, matrix, reinforcement material, or material origin [3]. The development of biomaterials drove a variety of biocomposite research, including materials that consist of the organic phase (silk fibroin (SF), collagen, hyaluronic acid (HA)) and inorganic phase (hydroxyapatite (HAp), amorphous calcium phosphate (ACP), and octacalcium phosphate (OCP)). To improve CaP properties, it can be combined with SF.

**Figure 1:**
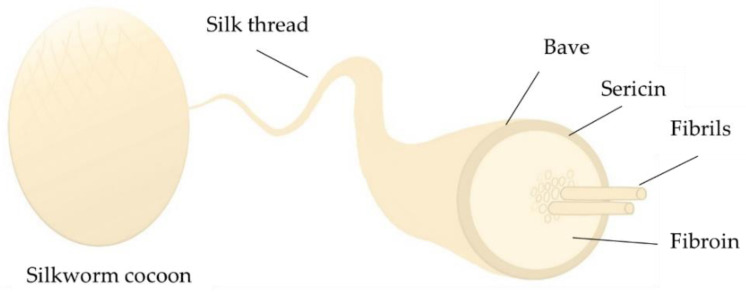
Composition of silkworm cocoon [4,5].

Silk fibroin is a widespread organic phase in composite materials. *Bombyx mori* silk is the most studied silk due to its outstanding properties. Although silk has been around for centuries, its primary use has been textile industry, but its use in medicine is quite new [4]. Silk’s main components are proteins, wax (in bave), sericin, and fibroin (Figure 1). 

**Figure 2:**
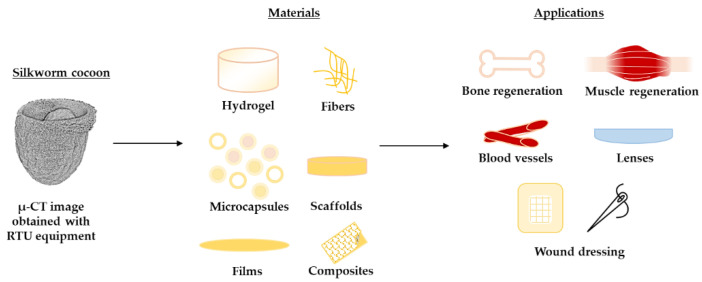
Silk fibroin application in medicine [6,7,8,9,10].

Fibroin is composed of three main proteinaceous components: heavy and light chain fibroin and glycoprotein. Heavy chain fibroin is hydrophobic, which consists of (Gly-Ala-Gly-Ala-Gly-Ser) pieces that have proven to increase silk’s crystallinity [6,7,8]. On the other hand, light chain fibroin with disulfide bonds is hydrophilic and elastic. The third component is a small glycoprotein, which is better known as sericin, it takes up to 30% of cocoons weight. Its main role is to hold the cocoon together [6,7]. Silk has two main conformations—silk I and silk II. Silk I has a helical, random coil structure, but silk II consists of β-sheets. Silk I is less crystalline compared to that of Silk II [7,8]. Silk is widely applicable in medicine, which is shown in Figure 2. Silk fibroin can be used as a drug delivery system in the form of hydrogels, microparticles, or scaffolds, but it can be used for bone regeneration as well [6,7,8,9,10]. Films and fibers can be used for wound dressing [11]. Silk fibroin is biocompatible and slowly degrades in the human body, which makes it the perfect matrix for drug delivery systems. By modifying SF concentration, crystallinity, and purity, its drug release kinetics can be adjusted [9,12]. 

Silk fibroin (SF) can be used in bone regeneration as a forming matrix, alongside calcium phosphates (CaP), because bones are not made from one material; they are a mix of organic and inorganic materials. SF/CaP composite materials have proved to provide bone regeneration [6,7,10,13]. SF/CaP composite materials can be synthesized by in situ method, which mimics the natural bone formation in the human body. CaP crystals can be easily incorporated inside SF polymer matrix due to their unique amino acid sequence, which provides a wide range of modifications [6]. Until now, CaP was synthesized in physiological salines [14], polypropylene fumarate [15], alginate [16], polyvinyl alcohol [17], chitosan [18], and collagen [18,19]. Only recently J. Mobika et. al. studied CaP synthesis in SF and concluded that SF increases composite material crystallinity and specific surface area [19]. By combining SF with CaP in situ, a better connection between the inorganic and organic phase can be made, as well as a material with promoted properties. 

**Figure 3:**
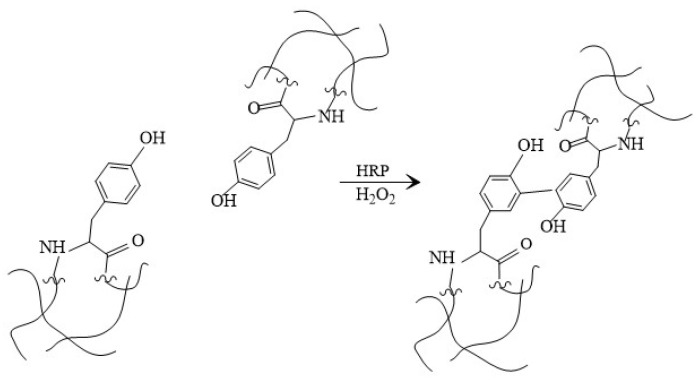
Schematic enzymatic polymerization of tyrosine residue inside SF.

As mentioned before, SF can be made into hydrogels, which can be used as drug delivery systems or implants. To enhance SF mechanical properties, gelatin is added. Gelatin (G) is an amphoteric, natural protein derived from collagen [20]. SF can be crosslinked physically or chemically. Physical crosslinking, such as electric current or heating, is safe, inexpensive, and less toxic. During this crosslinking process, ionic interactions occur, thus in chemical crosslinking covalent bonds are created, which are stronger than ionic bonds. Agents that are used in chemical crosslinking are glutaraldehyde and genipin, which create strong bonds, but are not that successful at cell toxicity tests. Enzymatic crosslinking is a subsection of chemical crosslinking. One type of enzymatic crosslinking is shown in Figure 3. To induce this crosslinking type the most used agents are horseradish perioxidase and peroxide (HRP)/H_2_O_2_, microbial transglutaminase (mTG), and mushroom tyrosinase. An advantage for enzymatic linking is that it can be easily made in mild aqua solutions, but it is an expensive method [8,9,21]. In the current work, HRP/ H_2_O_2_ is used as a crosslinker, because it is widely used, non-toxic, and creates a stable and elastic polymeric network [22]. 

In this study, we aim to synthesize SF/CaP composites at different pH values by in situ method. The pH values vary from 6 to 11 to achieve beta-tricalcium phosphate (β-TCP) at pH 6 and hydroxyapatite (HAp) at pH 11 [23]. Of these phases, β-TCP degraded faster in vivo than HAp [24]. At pH 8 and 10, it is planned to achieve a mixture of these phases. Obtained composites can be chemically crosslinked with G and HRP/H_2_O_2_ into hydrogels that work as controlled drug delivery systems. In the study, obtained hydrogels were used as drug delivery systems for controlled dexamethasone sodium phosphate (DEX) release. DEX is a water-soluble corticosteroid, which treats inflammation. It is used as a model active substance, for its ability to promote osteo-differentiation, bone regeneration, and cell proliferation [25,26]. The main goal of this research is to achieve SF/CaP composite by in situ method, which can be crosslinked with nontoxic agents and provide controlled drug release kinetics. 

## 2. Materials and Methods

Natural origin *Bombyx mori* silkworm cocoons were obtained from China. For silk degumming, Na_2_CO_3_ (Latvian Chemistry, Riga, Latvia) was used. CaCl_2_ (≥98%, Merck, Darmstadt, Germany) and ethanol (Latvian Chemistry, Riga, Latvia) was used to dissolve the degummed fiber, which was later dialyzed through cellulose membranes (flat width 25 mm 14 kDa, Sigma-Aldrich, St. Louis, MO, USA). SF with concentration 10.28 mg/mL ± 0.93 mg/mL was used, as well as calcium carbonate (Merck, Darmstadt, Germany) and phosphoric acid (75%, Latvian Chemistry, Riga, Latvia) for composite material synthesis. SF hydrogels were crosslinked with gelatin 100 mg/mL (from porcine skin, Fluka, Steinherm, Germany), 1000 U/mL HRP (≥250 U/mg, Sigma-Aldrich, St. Louis, MO, USA) and 245 mM H_2_O_2_ (30%, Merck, Darmstadt, Germany), where DEX (CHEMOS GmbH, Regenstauf, Germany) was added as a model drug. Drug release kinetics was studied in phosphate buffer saline (PBS, Sigma Aldrich, St. Louis, MO, USA). Cell cytotoxicity was tested using CCK-8 kit (Sigma Aldrich, St. Louis, MO, USA) and complete cell medium, which consisted of 1% penicillin-streptomycin (Sigma Aldrich, St. Louis, MO, USA), 10% calf serum (Sigma Aldrich, St. Louis, MO, USA), and 89% Dulbecco’s modified Eagle’s medium (Sigma Aldrich, St. Louis, MO, USA).

### 2.1. Preparation of SF

SF solution was prepared as described before [27]. Briefly, 3 g of *Bombyx mori* silkworm cocoons were degummed in 0.02 M Na_2_CO_3_ at 110 °C. After boiling, the obtained fiber was rinsed with water to wash out wax and sericin. The fiber was dried at room temperature for a week and then dissolved in CaCl_2_:H_2_O:C_2_H_5_OH (1:8:2). The obtained solution was dialyzed in deionized water for 7 days through cellulose membranes. The resulting SF concentration was determined with the evaporation method, where an empty micro test tube was weighted without SF, as well as with SF before and after drying at 60 °C for 3 days.

### 2.2. Calcium Phosphate In Situ Synthesis in the Silk Solution

**Figure 4:**
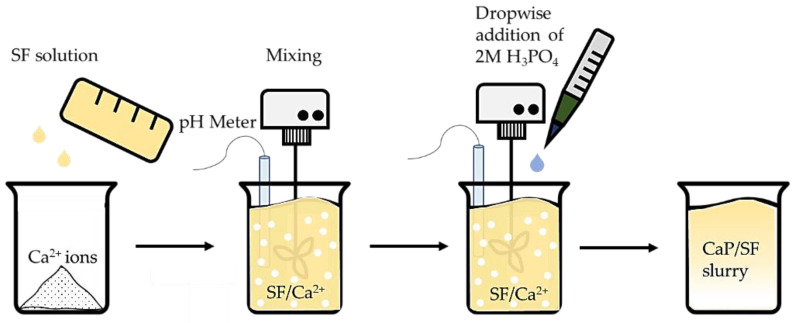
CaP/SF composite preparation.

CaP was synthesized in situ similar to HAp wet precipitation method [23], which is shown in Figure 4. 

Briefly, 0.56 g of CaO was added in 10.28 mg/mL ± 0.93 mg/mL SF solution and stirred at 150 rpm. While stirring, 2M H_3_PO_4_ was added dropwise and stirred at 300 rpm till pH 6, 8, 10, and 11. It was intended to achieve pure hydroxyapatite (HAp) and β-TCP and a mixture of both HAp and β-TCP. Three parallel replicates were performed for each synthesis. 

### 2.3. Silk Fibroin/Gelatin and CaP Hydrogel Preparation

Hydrogels were synthesized in situ after composite material preparation. 100 mg/mL of gelatin was added to the freshly made composite slurry and set aside to swell. After approximately 15 min it was dissolved by heating at 60 °C and stirring at 200 rpm. 1 mL of solution was transferred to each well in a 24-well plate, where firstly 10 μL of 1000 U/mL HRP and then 10 μL of 245 mM H_2_O_2_ was added. After mixing in the crosslinker, 5 mg DEX per well was added by gentle pipetting. The plate was let to crosslink in the incubator at 37 °C for 5 days. Hydrogels without CaP were prepared similarly, using freshly made SF solution as starting material, but skipping the step where CaP was made. Gelatin was added to SF and set to swell, later dissolved. A total of 1 mL of solution was transferred to each well, where HRP/H_2_O_2_ and DEX were added to SF/Gelatin mixture. 

### 2.4. Characterization of Obtained Samples

#### 2.4.1. Attenuated Total Reflection—Fourier Transformation Infrared Spectroscopy 

FT-IR spectra were recorded on Varian 800 FT-IR Scimitar series spectrometer (Varian, Palo Alto, CA, USA), using Attenuated Total Reflectance mode (PIKE Technologies, Madison, WI, USA) to identify functional groups in SF and SF/CaP composites. Before each measurement background spectra were subtracted from the sample spectrum. Sample spectra were recorded in the range of 4000 to 400 cm^−^^1^ with an accuracy of 4 cm^−^^1^ by performing 50 sample scans.

#### 2.4.2. X-ray Diffraction for CaP Composite Phase Analysis

XRD data were obtained on PANalytical X’pert PRO (Panalytical, Almeno, The Netherlands). X-ray anode: PW3373/00 Cu LFF DK 401991. Diffraction operating mode with radiation 40 kV and 30 mA was used according to the equipment instruction. Before analysis, the sample was fixed on a 27.0 mm silicon plate. The radiograph was taken in the 2θ 10°–70° range.

#### 2.4.3. Brunauer–Emmett–Teller Method

The surface area for lyophilized SF/CaP composites was determined using the Brunauer–Emmett–Teller method (BET). Equipment set for surface area analysis degasser Autosorb Degasser Model AD-9 (Quantachrome Instruments, Boynton Beach, FL, USA) and gas sorption system Quadrasorb SI (Quantachrome Instruments, Boynton Beach, FL, USA) and Quadra Win (Quantachrome Instruments, Boynton Beach, FL, USA) was used. Prior to the analysis, the samples were degassed to free the surface from moisture and other impurities that are sorbed at the surface of the material. For degassing 9 mm round sample cells are used, degassing time is 24 h at room temperature. The specific surface area of the samples was analyzed using the gas sorbtion system Quadrasorb SI from low-temperature (77 K) nitrogen physical absorption-desorption isotherms.

#### 2.4.4. SF/CaP Hydrogel Gel Fraction and Swelling Degree

Gel fraction describes the insoluble part of the hydrogel. It was determined by immersing dried prepared hydrogels and composite hydrogels in 20 mL of deionized water for 48 h in an incubator at 37 °C and stirring at 100 rpm [28]. After immersion, the hydrogels were taken out of the liquid, lyophilized, and weighed. Gel fraction was calculated by the following Equation (1):

(1) $$W_{GF}\;=\;\left(\frac{W_{d}}{W_{i}}\right)\;\times \;100\;\left(\%\right),$$

where *W_d_* and *W_i_* are the mass of dried sample and mass of insoluble part of the hydrogel after lyophilization, respectively. 

The swelling degree was determined by weighting composite hydrogel after a certain amount of time [28,29]. Hydrogels were taken out of the water after 1; 2; 3; 4; 5; 6; 24 h, and the excess water on the surface was dried on a wet paper tissue. The swelling degree was calculated according to Equation (2):

(2) $$W_{SD}\;=\;\left(\frac{W_{S}\;-\;W_{d}}{W_{d}}\right)\;\times \;100\;\left(\%\right),$$

where *W_d_* and *W_s_* are the mass of dried sample and mass of the swelled sample after each amount of time. 

#### 2.4.5. Hydrogel Drug Release

The drug release kinetics was studied using UV/VIS spectrophotometer Evolution 300 (Thermo Fisher, Waltham, MA, USA) at 242 nm. The calibration curve was constructed by analyzing the different concentrations of DEX standards diluted in PBS [30]. The standards and the first analyzing solutions were analyzed the same day. Precisely 8 mg of DEX was diluted in 100 mL PBS. Standard concentrations varied from 0.8 μg/mL to 80 μg/mL. Firstly, the samples with DEX and blank samples were immersed in 20 mL PBS solution in an incubator at 37 °C and stirred at 100 rpm. After 1; 2; 4; 6; 24; 48; 72 h 5 mL of each sample was taken out for analysis and 5 mL of PBS was returned to the container, keeping the total dissolution medium volume constant. After the data were obtained, drug release kinetics were calculated.

#### 2.4.6. Micro-Computed Tomography (μ-CT)

Prior to micro-computed tomography (microCT) scanning, hydrogels were lyophilized. Samples scans were made using cabinet cone-beam microCT (µCT50, Scanco Medical AG, Bruttisellen, Switzerland) settings: 70 kVp energy; 114 μA tube current; 1500 ms integration time and 10 µm voxel size). Each sample was scanned for approximately 7h. Reconstruction of 3D datasets from microCT projection data, including beam hardening correction, was performed automatically after completion of each cone beam image stack. The visualization module performs complex 3D data reproduction for large data sets using high-quality beam tracking algorithms. 

#### 2.4.7. Scanning Electron Microscopy 

To visualize the surface morphology of the synthesized composites and hydrogels, Tescan Mira/ LMU (Tescan, Brno, Czech Republic) electron microscope was used. Firstly, the samples were attached to aluminum pin stubs with conductive carbon tape, and before analysis, the samples were sputter-coated with a thin layer of gold at 25 mA for 3 min using Emitech K550X (Quorum Technologies, Ashford, Kent, UK). Secondary electrons created at 5 kV were used to characterize both composites and hydrogels.

#### 2.4.8. Cell Cytotoxicity Tests

Prior to sample testing on cells, water vapor sterilization at 105 °C was performed with electronic tabletop autoclave ELARA 11 (Tuttnauer, Breda, The Netherlands). Before sterilization, samples were packed in sterilization packing material using HN 850 DC rolling mill (Hawo, Obrigheim, Germany). 

The cytotoxicity of the obtain hydrogels was tested by using CCK-8 kit and mouse fibroblast 3T3 cells. At first 2.5 × 10^4^ cells per well were seeded in 1 mL of full cell medium in 24-well plates and left in the incubator for 24 h (5% CO_2_, 37 °C). After a day the medium was changed, and sterilized pieces hydrogels were put onto cells and put back in the incubator for another 24 h. Later, after completing the one-day incubation samples were taken out of the wells and 50 μL of CCK-8 solution was added to the cells at each well. The plates were incubated for 2 h, followed by absorbance measurement at 450 nm with Infinite M Nano microplate reader (Tecan, Männedorf, Switzerland). Each sample had three replicates, positive control (cells and medium) and medium as a background measurement. 

Cell viability was calculated by the following Equation (3):

(3) $$CV\;=\;\frac{\left(OD_{S}\;-\;OD_{BG}\right)}{\left(OD_{C}\;-\;OD_{BG}\right)}\;\times \;100\%$$

where *CV*—cell viability, *OD_S_*, *OD_C_* and *OD_BG_*—optical density for the sample, control, and background. 

### 2.5. Statistic Analysis

All the obtained results were represented with mean value ± standard deviation (SD). To evaluate the significance of the results, Student’s materials-14-07191nce *p*  < 0.05. One-way and two-way variance analysis (ANOVA) was performed to assess the obtained results.

## 3. Results

### 3.1. Raw Silk Fibroin Characterization

**Figure 5:**
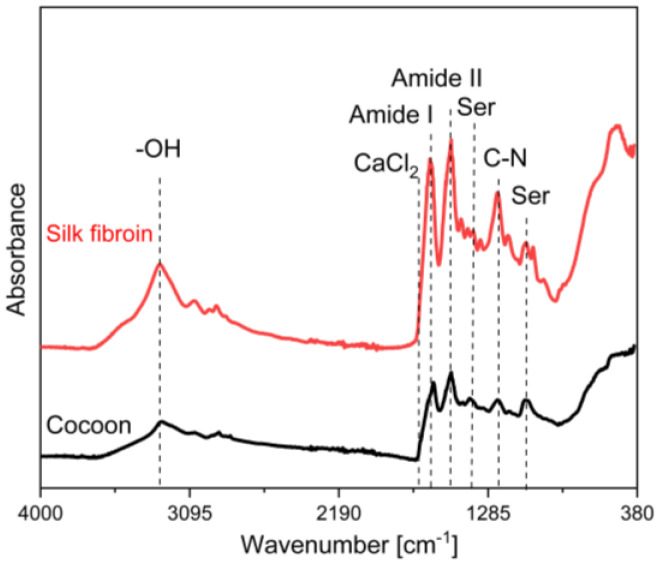
FT-IR spectra of *Bombyx mori* cocoon and obtained SF solutions.

Silk fibroin FT-IR ATR spectra is shown in Figure 5, which shows data from 4000 to 380 cm^−^^1^. The thin SF films after evaporation were examined to determine functional groups.

The two main factors that were examined in the spectra were whether there was present sericin (Ser) and CaCl_2_. Sericin levels weren’t determined with this method after degumming. No characteristic maximums for sericin in 1050 cm^−^^1^ or 1400 cm^−^^1^ were found. A typical peak in 1657 cm^−^^1^ for CaCl_2_ is not observed [27,31,32,33]. 

Regarding functional groups, Table 1 presents band assignments. The broad peak at 3270 cm^−^^1^ shows the persistence of water, which indicated O-H band stretching. Amid group maximums were detected at 1650 cm^−^^1^ (Amide I C=O), 1520 cm^−^^1^ (Amide II N-H), 1235 cm^−^^1^ (Amide III, C-N) [16,31,33,34].

### 3.2. SF/CaP Composite Characterization

**Figure 6:**
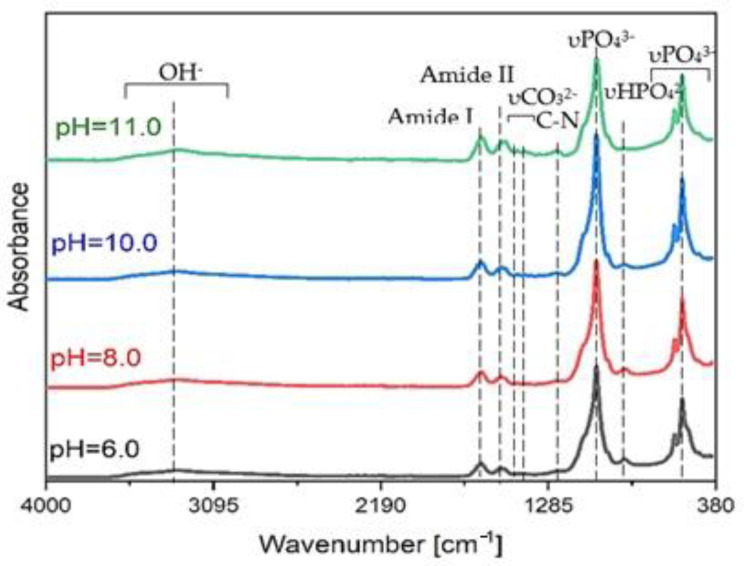
FT-IR spectra of CaP/ SF and composites at different pH.

FT-IR ATR spectroscopy can be used to study CaP incorporation in SF. Spectra of the prepared samples are shown in Figure 6. The amide groups—amide I (C=O), amide II (N-H), and amide III (C-N) were found at 1624, 1518, and 1243 cm^−^^1^ respectively in the SF/CaP composite materials, which indicated that CaP was incorporated in SF [16], but they have slightly shifted due to Ca^2+^ persistence, which is another indicator of a successful synthesis. The biggest shift can be observed for amide I that means that the SF structure has concerted to β-conformation from random coal [16]. Amide groups for pure SF can be found at 1625, 1520, and 1235 cm^−^^1^. The OH^-^ band vibrations are present in all samples in approximately 3270 cm^−^^1^, including synthesized composites, which stage the persistence of water. Quite a small peak is present in 1421 cm^−^^1^ for CaP and composites due to the CO_3_^2−^ group. That means that phosphates have reacted with air (CO_2_) or β-type carbonated apatite is present, which is not significant because β-carbonated apatite is in the natural bone as well [16,35,36].

**Figure 7:**
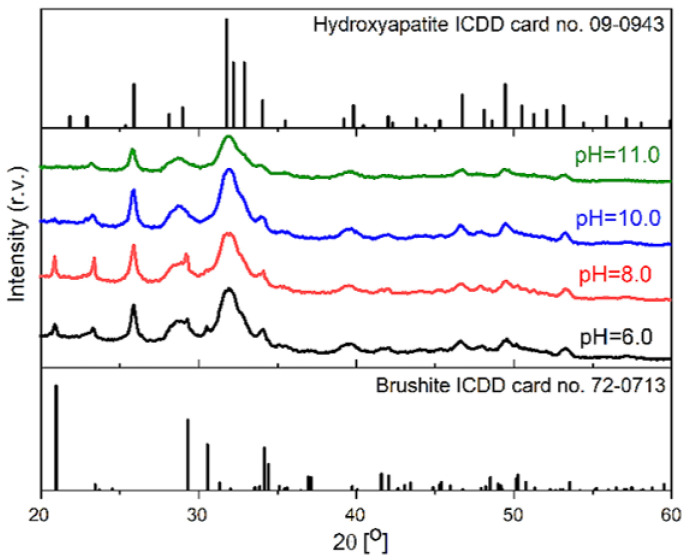
XRD pattern of synthesized composites in SF.

In this study, CaP was obtained in different pH values. In Figure 7, XRD patterns of CaP from pH 6 to pH 11 are shown. All the acquired composites are more amorphous because diffuse diffraction reflexes can be observed. The obtained spectra for each pH values are quite similar, but it changes due to different pH value. From pH 6 to 8, brushite (ICDD card no. 11-0293) is present in the composites. Characteristic brushite peaks at 20.9°, 29.3°, and 30.5° are present with a mix with hydroxyapatite (ICDD card no. 09-0432), but it decreases at higher pH values. As we can see brushite is no longer present, and when pH is 10 or 11, pure hydroxyapatite (HAp) phase in SF solution is obtained. Typical HAp diffraction reflexes can be seen at 31.7°, 32.2°, and 32.9°.

**Figure 8:**
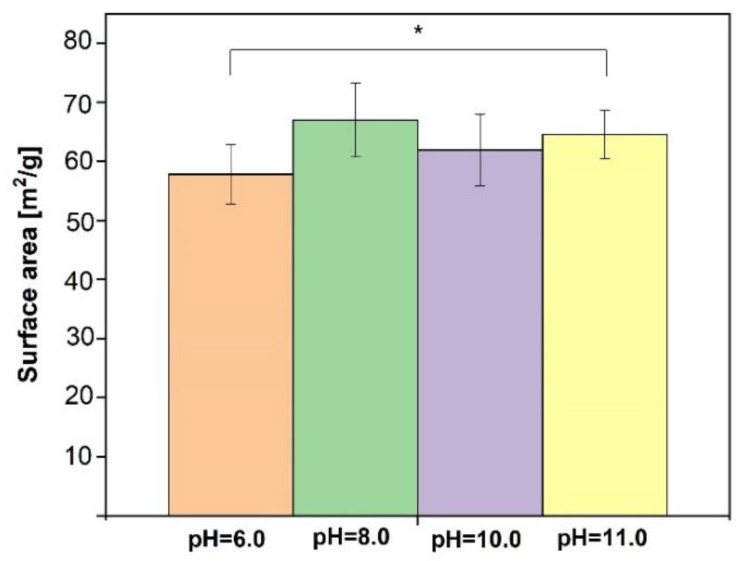
Specific surface area of synthesized composites in SF (*n* = 3, * *p* > 0.05).

Specific surface area (SSA) analysis was performed on lyophilized SF/CaP composite slurries to evaluate how it changes at different CaP phases. Figure 8 shows that all the composites have a similar SSA, which means that pH values (type of CaP) do not affect the composite material SSA. According to Student’s *t*-test, the obtained data do not indicate a statistically significant difference, because Levene’s *p*-value is above statistical significance 0.05. Comparing SF/CaP composite SSA to pure HAp obtained in water, which is 94.9 [37], shows that the SSA is lower, which is due to SF presence. 

**Figure 9:**
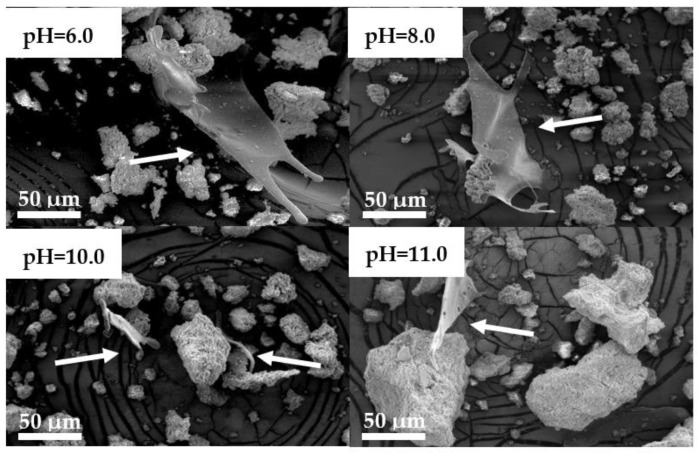
SEM images of SF/CaP composites synthesized at different pH values.

SEM was used to characterize the freeze-dried SF/CaP composite materials. SF is present in all the composites (Figure 9), due to observed fibrils, which are evenly spread throughout the sample. Particle size was measured with “ImageJ” software. The smallest particle size was observed for composite which consisted of both hydroxyapatite and brushite from 6.5 to 50.7 μm (pH = 8), but the largest particles were for the composite with mainly brushite (pH = 6) from 7.8 to 71.3 μm. Composites synthesized at pH = 10 and 11 particles were from 7.1 to 65.0 μm and from 6.6 to 75.6 μm, respectively. Particle size does not change between the different phases of CaP. 

### 3.3. SF/G/CaP Hydrogel Properties

#### 3.3.1. Gel fraction

**Figure 10:**
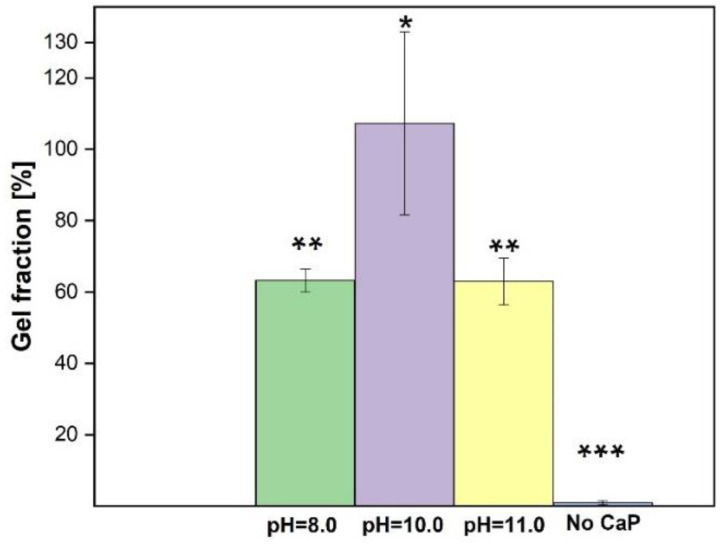
Gel fraction for SF/CaP/G/HRP hydrogels (*n* = 3, * *p* < 0.05, ** *p* > 0.05, and *** *p* < 0.01).

Gel fraction (GF) of synthesized blank hydrogels is similar in all the synthesized materials, as it is represented in Figure 10. 

Samples at pH 6 form an unstable hydrogel, which starts to dissolve after 2 h, and therefore it was not possible to obtain a one-piece sample after 48 h. The reason for this might be too low a pH value. At lower pH values, HRP becomes inactive. As HRP is the crosslinker in our research, therefore the gel fraction changes in between the samples [38]. Gel fraction of the hydrogels synthesized at pH 8 and pH 11 do not statistically differ, which means that CaP type is not the dominant factor that affects how much of insoluble part the hydrogel has. Statistical analysis of the data shows that gel fraction between hydrogels synthesized at pH 8 and pH 11 does not have a significant difference; only hydrogels synthesized at pH 10 and without CaP have a significant difference in gel fraction results.

#### 3.3.2. Swelling Degree

**Figure 11:**
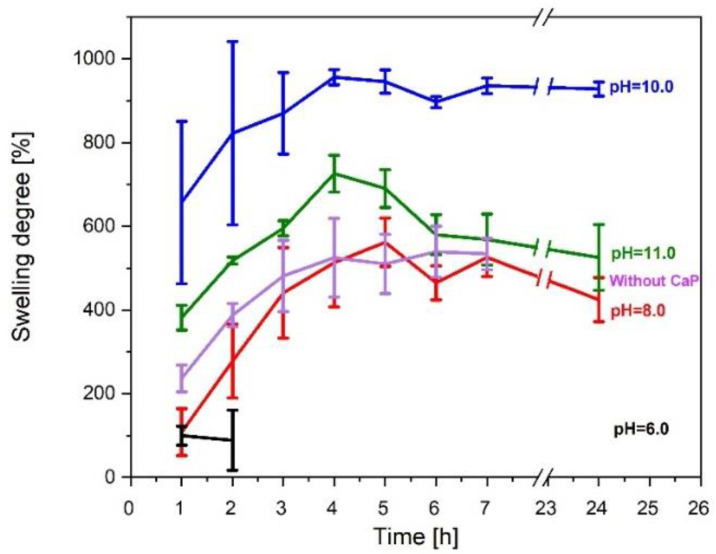
Swelling degree of SF/CaP/G/HRP hydrogels.

One of the most important properties of hydrogel is its ability to absorb water. The results in Figure 11 show that the swelling degree is higher in those hydrogels, where hydroxyapatite is present. 

However, hydrogels containing brushite have a relatively lower swelling degree. On the other hand, samples without any kind of CaP swell at first but then degrade after 7 h due to lower gel fraction. Hydrogels made at pH 6 start to dissolve after 2 h, which is due to weak linkage inside hydrogels, however, hydrogels without CaP started to degrade after 24 h but swelled for the first 7 h. The degradation is due to low gel fraction. 

#### 3.3.3. Drug Release Kinetics

**Figure 12:**
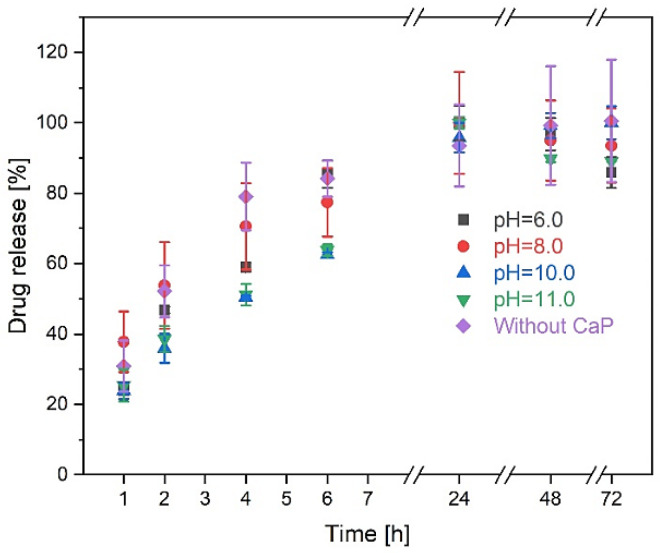
DEX drug release kinetics for SF/CaP/G/HRP hydrogels.

Hydrogel drug release properties were evaluated (Figure 12), after hydrogels were incubated for 1; 2; 4; 6; 24; 48; 72 h. 

Burst release is different in between the samples. Hydrogels, which contained brushite had burst release after 2 h, where 38% (pH 6) and 53% (pH 8) were released. On the other hand, samples with HAp rapidly released the drug after 4 h—50% (pH 10) and 51% (pH 11) of the total drug.

The obtained results show that drug release kinetics was not affected by the CaP phase. The drug is completely released from hydrogels after 24 h, except the one that is made in pH 10, which completely released the drug after 72 h. This could be linked to a high gel fraction, as it is 20–40% higher than other samples. 

Hydrogel without CaP showed just as good drug release properties as that of hydrogels containing CaP. Complete drug release was acquired after 24 h. 

#### 3.3.4. SF/G/CaP Hydrogel Morphology

**Figure 13:**
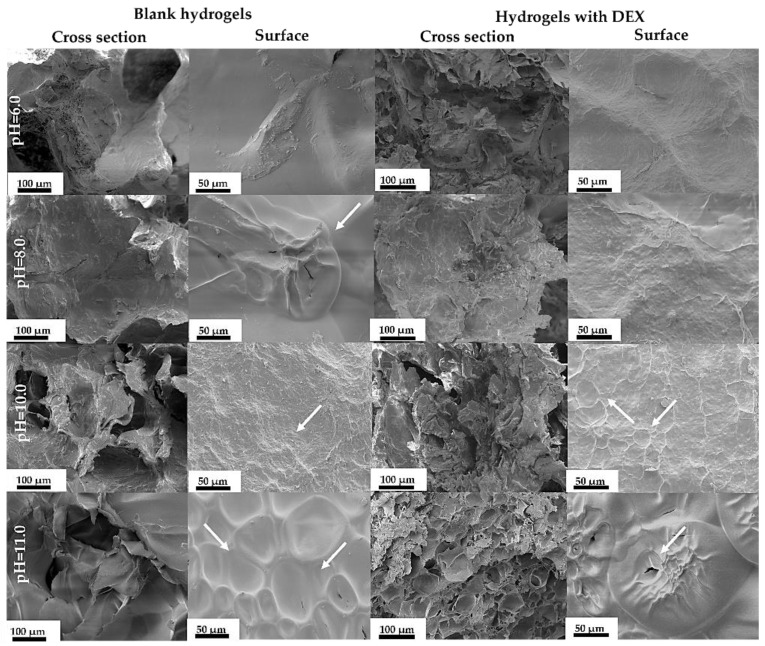
SEM images of blank and DEX-loaded hydrogel surface and cross section morphology.

To get a better understanding of lyophilized hydrogel surface and cross section, their morphology was studied (Figure 13).

**Figure 14:**
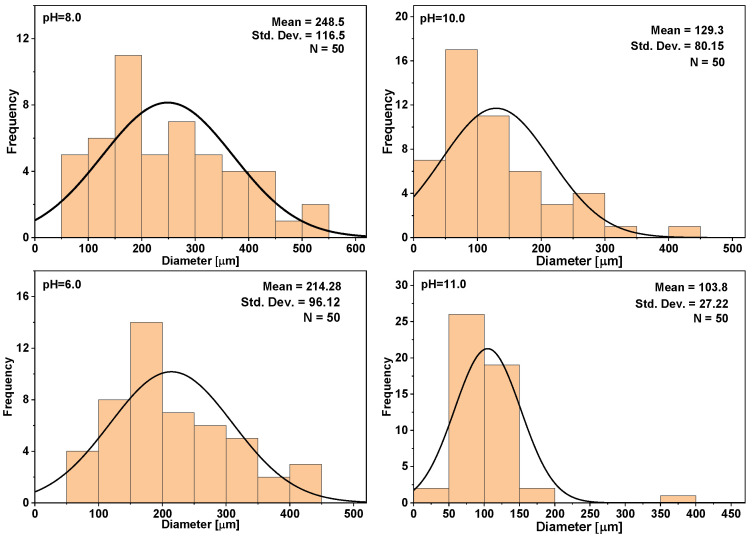
Pore distribution in drug-loaded hydrogels.

Surface morphology is different in between the hydrogels. All blank samples without drugs from pH 8 to pH 11 have a porous surface, but the blank sample at pH 6 does not have any pores. In hydrogel cross section a porous structure can be observed. The long open pores can be observed due to lyophilization, which stabilized the whole system [38]. The pore size changes in hydrogels, which indicates a heterogeneous structure; the pores are not evenly distributed (Figure 14).

DEX-loaded hydrogels with pure hydroxyapatite (at pH 10) have outward pores, which cannot be seen in other samples. On the other hand, drug-loaded hydrogels synthesized at pH 11 (Figure 12) have the smallest pores, which cannot be seen in other scaffolds. The smaller the pores, the less water or gas the sample can take up, because the structure is denser.

**Figure 15:**
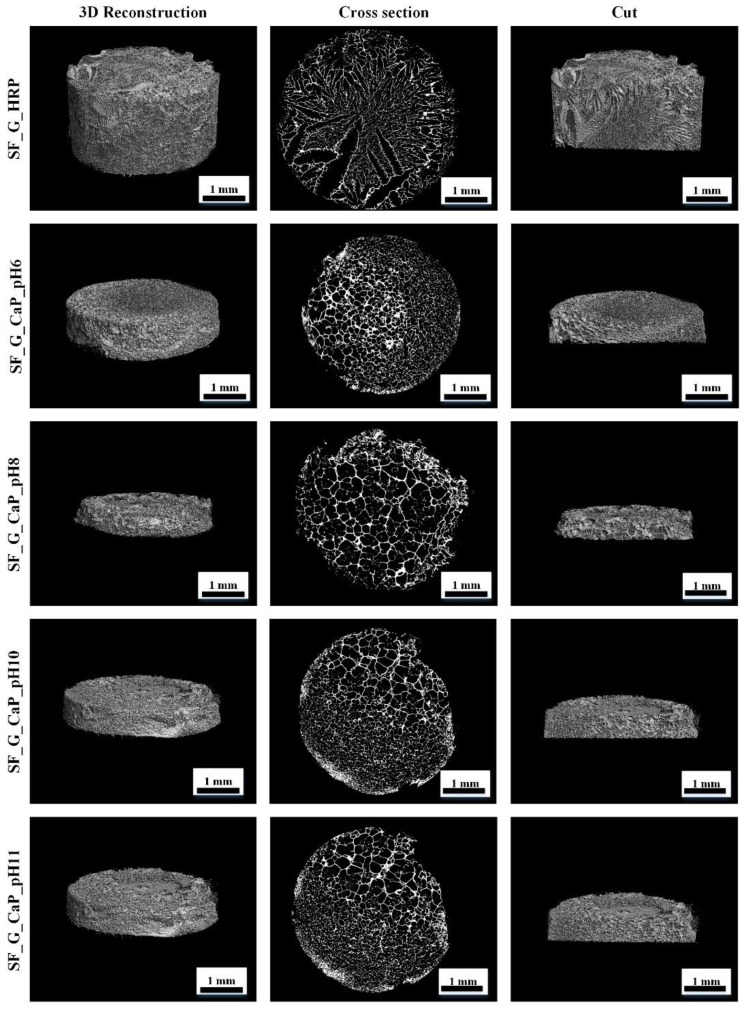
Hydrogel 3D structure.

After examining hydrogel 3D structure, an uneven pore distribution can be observed in Micro-CT images (Figure 15). One reason for that is the freezing process. If the hydrogel is slowly frozen the water inside pores can grow slowly, thus freezing with nitrogen freezes the sample instantly. 

The main reason for uneven pore apportionment can be linked with freshly synthesized composites, which have an unequal particle size disposal. This leads to uneven pore distribution inside the hydrogel. 

#### *3.3.5.* Cell Toxicity

**Figure 16:**
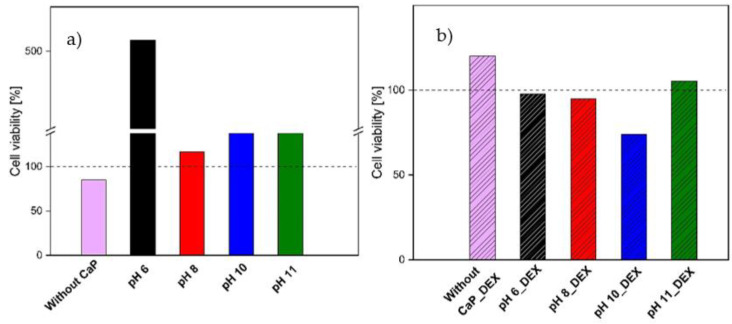
SF/G/HRP and SF/CaP/G/HRP hydrogel cytotoxicity after 24 h (**a**) without DEX and (**b**) with DEX (Means ± SD, *n* = 3).

The cellular cytotoxicity results obtained with CCK-8 kit are shown in Figure 16.

The obtained results show that neither SF/G/HRP hydrogels nor SF/CaP/G/HRP samples are cytotoxic. After result evaluation, drug-loaded samples showed higher cytotoxicity, than blank samples. Overall, samples with brushite, synthesized at pH 6, show the highest cell viability on the contrary, the lowest viability is for the samples without CaP. Comparing drug loaded samples, the highest toxicity was found in the samples with HAp (pH 10), but the lowest was in the sample without CaP. 

There are significant differences between samples with and without drugs (*p* < 0.05), but if each pH is evaluated separately, then the only significant difference can be observed for pH 6 (*p*  < 0.05).

## 4. Discussion

This study examined a new method for creating in situ synthesized SF/CaP composites and SF/G/CaP/HRP hydrogel drug delivery systems. To date, no one synthesized SF/CaP composites in situ from pH 6 to pH 11. This pH range shows the formation of different CaP phases in silk solution. These composites have a high potential of becoming one of the best new age biomaterials, due to their interaction between organic and inorganic phases that mimics the natural CaP synthesis inside the human body. By varying the CaP phase synthesized in SF, different composites and hydrogels can be obtained. The most valuable properties such as gel fraction, swelling degree, and drug release can be observed for the hydrogels, which are synthesized at pH 10 and consisted of silk fibroin and hydroxyapatite. 

The SF/CaP composite and SF/CaP/G/HRP hydrogel structural, phase, surface properties, and drug release kinetics were examined. Different types of CaP can be synthesized in SF at different pH values. Composites from pH 6 to 8 contain brushite and HAp, however from 10 to 11 pure HAp can be achieved. There is a slight notice of carbonate ions in higher pH value synthesized CaP, but it is not a flaw, because each bone regeneration study needs a specific CaP phase [39,40]. The interaction between SF and CaP happens on account of carboxyl groups, which are present in SF. They ensure a nucleation center for calcium ions, which bind phosphate ions around themselves. 

The acquired composite suspensions and pure SF/G were crosslinked with HRP/H_2_O_2_ and loaded with DEX. Gelatin provides a denser, more elastic polymer network [40]. The obtained hydrogels showed a remarkable swelling degree, but not as good overall stability. Hydrogels containing CaP started to degrade after 24 h except the hydrogel made at pH 6, which started to degrade after 2 h, but without CaP started to degrade after 7 h, which leads to the conclusion that CaP stabilizes the hydrogel network at pH values above pH 6. Differences between replicates occur due to the draining process, which is done before weighting. The achieved hydrogels are very adhesive and attach to the wet paper tissue and a part of the hydrogel is left on the tissue. The adhesion arises from the contained tyrosine groups in SF and gelatin [41,42]. All and all, hydrogels, which contain HAp are more stable and have a higher swelling degree. This can be deducted after gel fraction experiments, where samples with CaP showed 50 to 80 times higher gel fraction than samples without CaP. The same results can be seen in Ribeiro et. al. work on SF/nanohydroxyapatite composite hydrogels, where hydrogels swelled 500% water of their dry mass [13]. Conversely, SF/polyacrylamide/graphene oxide hydrogels had an even higher gel fraction till 1200% [9]. Obtained SF/HAp/G hydrogels showed a comparable swelling rate to polymer with synthetic origin (956.32% ± 18.20%).

The type of CaP does influence hydrogel properties. Hydrogels, which contained brushite, showed faster degradation and lower insoluble mass percent, contrarily samples with HAp were more stable and had higher gel fractions. Overall gel fraction is higher if Ca^2+^ ions are included in the hydrogel because Ca^2+^ ions make the polymer matrix more stable by creating an electrostatic interaction with silk and gelatin [43]. The polar and charged side chain groups inside the silk structure work as nucleation centers for CaP [16]. Close bonding between the inorganic and organic phases might increase the stability of hydrogel. On the other hand, the lack of stability inside the SF/G/HRP hydrogel is due to weak crosslinking between the tyrosine groups inside silk fibroin [41].

The drug release kinetics confirmed the previous observations. The more stable the system—the longer it takes for the drug to completely excrete from the polymer network. Hydrogels without CaP released 84.19% ± 5.20% drug after 6 h, a similar pattern is observed for the hydrogels made at pH 6, where 85.33% ± 3.88% drug is released after 6 h. Both samples completely released the active substance after 24 h. These hydrogels have the lowest gel fraction, which means that gel fraction affects drug release kinetics. On the other hand, hydrogels with pure HAp excreted only approximately 62% of the overall included drug after 6 h, thus complete drug release was observed after 24 h for pH 11 and 72 h for hydrogels made at pH 10. Acute inflammatory response accrues after 24–48 h [44], which means that our hydrogels could be suitable to provide the needed medication at the most important inflammation starting stage. Comparing our SF/CaP/G/HRP hydrogels to other DEX-containing hydrogels, drugs are excreted faster than from other hydrogel matrice. DEX can be sustainably released from PVA hydrogels in 25 days [45], but from PLGA-PEG-PLGA hydrogels, it was released in 350 h (approximately 14 days) [46]. However, if the hydrogel contained β-TCP the drug was fully released after 72 h [47].

Not only bonds, phases, and drug release kinetics were studied for these materials—the surface was characterized as well. The achieved data showed that specific surface area does not change in the terms of CaP type, but the particle size is similar between the obtained composites and after SEM analysis; therefore, SF is present in all the composites. Hydrogel pore size is different for each hydrogel. The smallest pores are for the hydrogel, which was synthesized at pH 11 and the biggest pores had the hydrogel, which contained both hydroxyapatite and brushite. SF/CaP/G/HRP hydrogels have a similar porosity to other organic/inorganic phase hydrogels, for example, Scgelovs et al. achieved well organized 20–200 μm porosity in ε-polylysine/hyaluronic acid/hydroxyapatite hydrogels [48]. Kim et al. synthesized SF/HAp hydrogels contained even pore distribution, with pore size from 130–250 μm, average pore size—161 ± 42 μm [49], therefore our obtained hydrogels had smaller average pore size—80 ± 38 μm. On the other hand, Nie et al. chitosan/gelatin/ biphasic calcium phosphate had pores from 10 to 200 μm. The pore size matters for tissue engineering. Each cell needs a unique pore size. For example, for the bone to regenerate, pores need to be from 100–350 μm, thus osteoid needs even smaller pores: 40–100 μm [50]. All in all, SF/CaP/G/HRP hydrogels would be suitable for bone regeneration due to their pore size. 

To ensure that obtained hydrogels could be used for medical application, in vitro cell viability tests were performed. The tests confirmed that hydrogels are nontoxic and could proceed further with additional in vitro and in vivo assays. However, there is a significant difference between the samples with CaP and without as well as samples with and without drugs having significantly different cell viability results. Sterilized samples without CaP and drugs showed lower viability (Cell viability > 70%). On the other hand, samples with DEX show slightly higher cytotoxicity compared to samples without drugs. Hydrogel with brushite (pH 6 and pH 8), show cell viability above 95%. Hydrogel with DEX and hydroxyapatite (pH 10) showed lower viability (Cell viability > 70%). The reason why samples with drugs show higher toxicity is that DEX might react with calcium ions and change its coordination [51]. Comparing blank samples with and without CaP, CaP increases 3T3 fibroblast cell proliferation, thus leftover sericin in samples without CaP might be the reason that these samples show lower viability [52]. In previous studies, Lie et al. has concluded that SF does not have adverse effect on fibroblast growth [53] and the same results can be concluded in our research. Silk fibroin has a positive effect on growth factor expression, which can improve angiopoietins [53]. Unfortunately, growth factor expression was not studied in our research, but it could be included in future studies. Calcium ions can improve cell proliferation [54,55], however too much calcium ion concentration can enhance cell death [56,57]. Looking at the results obtained from different types of CaP-containing phases in the samples, the sample with brushite (pH 6) shows the highest cell viability, which might be due to Ca^2+^ release. Klammer et al. studied brushite-containing scaffolds, which showed high cell viability as well [58], although Z. Shi et al. also concluded that HAp does improve cell proliferation, however particle size and type is the key factor for cell survival [59]. In future studies, cells could be encapsulated to fully ensure bone regeneration as the sample preparation has mild crosslinking conditions.

## 5. Conclusions

CaP can be incorporated in SF by in situ synthesis, which provides organic and inorganic phase interaction. By changing the pH of the solution, different CaP can be synthesized. Composites from pH 6 to 8 consist of HAp and brushite, contrarily composites from pH 10 to 11 consist of pure HAp. The specific surface area does not change with the change of phase, which is due to low SF concentration. 

Our results show that enzymatically crosslinked hydrogels containing CaP have a better swelling degree, gel fraction, and drug release kinetics. The highest swelling degree and gel fraction were for the hydrogel that was made in pH 10 and contained pure HAp, but the lowest was for the hydrogels that contained both—HAp and brushite. CaP increases gel fraction and swelling degree because hydrogels that do not contain CaP have remarkably lower gel fraction. The drug release kinetics slightly differ in terms of CaP. The sample which contained pure HAp at pH 10 released the active substance slower (after 72 h) than any other sample (all the rest after 24 h). Hydrogels with CaP showed higher cell viability than hydrogels without CaP.

Our research proves that the obtained hydrogels could be suitable for medical application due to their composition, drug release kinetics, and biocompatibility.

## Figures and Tables

**Table 1:** Main bands present in SF from 4000 to 380 cm^−1^.

Position (cm^−1^)	Assignment	Reference
3270	*v* OH^−^	[16]
1657	CaCl_2_	[34]
1650	Amide I	[31,34]
1520	Amide II	[31,34]
1400	Ser	[33]
1235	*v* C-N	[31,34]
1070	Ser	[33]

*v*—stretching.

## Data Availability

The data presented in this study are available on request from the corresponding author.

## References

[1] Lin X.-L., Gao L.-L., Li R., Cheng W., Zhang C.-Q., Zhang X. (2019). Mechanical Property and Biocompatibility of Silk Fibroin–Collagen Type II Composite Membrane. Mater. Sci. Eng. C.

[2] World Health Organization (WHO) (2015). World Report of Ageing and Health.

[3] Rajak D.K., Pagar D.D., Kumar R., Pruncu C.I. (2019). Recent Progress of Reinforcement Materials: A Comprehensive Overview of Composite Materials. J. Mater. Res. Technol..

[4] Ude A.U., Eshkoor R.A., Zulkifili R., Ariffin A.K., Dzuraidah A.W., Azhari C.H. (2014). Bombyx Mori Silk Fibre and Its Composite: A Review of Contemporary Developments. Mater. Des..

[5] Cao T.T., Zhang Y.Q. (2016). Processing and Characterization of Silk Sericin from Bombyx Mori and Its Application in Biomaterials and Biomedicines. Mater. Sci. Eng. C.

[6] Ma D., Wang Y., Dai W. (2018). Silk Fibroin-Based Biomaterials for Musculoskeletal Tissue Engineering. Mater. Sci. Eng. C.

[7] Hardy J.G., Scheibel T.R. (2010). Composite Materials Based on Silk Proteins. Prog. Polym. Sci..

[8] Johari N., Moroni L., Samadikuchaksaraei A. (2020). Tuning the Conformation and Mechanical Properties of Silk Fibroin Hydrogels. Eur. Polym. J..

[9] Galateanu B., Hudita A., Zaharia C., Bunea M.-C., Vasile E., Buga M.-R., Costache M. (2019). Silk-Based Hydrogels for Biomedical Applications. Polym. Polym. Compos. A Ref. Ser..

[10] Xu Z., Shi L., Yang M., Zhu L. (2019). Preparation and Biomedical Applications of Silk Fibroin-Nanoparticles Composites with Enhanced Properties—A Review. Mater. Sci. Eng. C.

[11] Patil P.P., Reagan M.R., Bohara R.A. (2020). Silk Fibroin and Silk-Based Biomaterial Derivatives for Ideal Wound Dressings. Int. J. Biol. Macromol..

[12] Wani S.U.D., Gautam S.P., Qadrie Z.L., Gangadharappa H.V. (2020). Silk Fibroin as a Natural Polymeric Based Bio-Material for Tissue Engineering and Drug Delivery Systems-A Review. Int. J. Biol. Macromol..

[13] Ribeiro M., De Moraes M.A., Beppu M.M., Garcia M.P., Fernandes M.H., Monteiro F.J., Ferraz M.P. (2015). Development of Silk Fibroin/Nanohydroxyapatite Composite Hydrogels for Bone Tissue Engineering. Eur. Polym. J..

[14] Tas A.C. (2014). The Use of Physiological Solutions or Media in Calcium Phosphate Synthesis and Processing. Acta Biomater..

[15] Hakimimehr D., Liu D., Troczynski T. (2005). In-Situ Preparation of Poly (Propylene Fumarate)—Hydroxyapatite Composite. Biomaterials.

[16] Mobika J., Rajkumar M., Nithya Priya V., Linto Sibi S.P. (2020). Substantial Effect of Silk Fibroin Reinforcement on Properties of Hydroxyapatite/Silk Fibroin Nanocomposite for Bone Tissue Engineering Application. J. Mol. Struct..

[17] Timofejeva A., D’Este M., Loca D. (2017). Calcium Phosphate/Polyvinyl Alcohol Composite Hydrogels: A Review on the Freeze-Thawing Synthesis Approach and Applications in Regenerative Medicine. Eur. Polym. J..

[18] Lawson A.C., Czernuszka J.T. (1998). Collagen-Calcium Phosphate Composites. J. Eng. Med..

[19] Lin X., Li X., Fan H., Wen X., Lu J., Zhang X. (2004). In Situ Synthesis of Bone-like Apatite / Collagen Nano-Composite at Low Temperature. Mater. Lett..

[20] Jaipan P., Nguyen A., Narayan R.J. (2017). Gelatin-Based Hydrogels for Biomedical Applications. MRS Commun..

[21] Kengla C., Lee S.J., Yoo J.J., Atala A. (2019). 3-D Bioprinting Technologies for Tissue Engineering Applications. Rapid Prototypign Biomater..

[22] Zhou B., Wang P., Cui L., Yu Y., Deng C., Wang Q., Fan X. (2017). Self-Crosslinking of Silk Fibroin Using H2O2-Horseradish Peroxidase System and the Characteristics of the Resulting Fibroin Membranes. Appl. Biochem. Biotechnol..

[23] Salma K., Berzina-Cimdina L., Borodajenko N. (2010). Calcium Phosphate Bioceramics Prepared from Wet Chemically Precipitated Powders. Process. Appl. Ceram..

[24] Daculsi G., Legeros R.Z., Nery E., Lynch K., Kerebel B. (1989). Transformation of Biphasic Calcium Phosphate Ceramics In Vivo: Ultrastructural and Physicochemical Characterization. J. Biomed. Mater. Res..

[25] Kim Y.H., Tabata Y. (2015). Dual-Controlled Release System of Drugs for Bone Regeneration. Adv. Drug Deliv. Rev..

[26] Wu C., Miron R., Sculean A., Kaskel S., Doert T., Schulze R., Zhang Y. (2011). Proliferation, Differentiation and Gene Expression of Osteoblasts in Boron-Containing Associated with Dexamethasone Deliver from Mesoporous Bioactive Glass Scaffolds. Biomaterials.

[27] Egle K., Dubnika A. (2020). Development of Bioadhesive Biomaterials Based on Silk and Hyaluronic Acid. Key Eng. Mater..

[28] Tsihlis N.D., Murar J., Kapadia M.R., Ahanchi S.S., Oustwani C.S., Saavedra J.E., Keefer L.K., Kibbe M.R. (2010). Hydrogels: Methods of Preparation. J. Vasc. Surg..

[29] Qu X., Yan L., Liu S., Tan Y., Xiao J., Cao Y., Chen K., Xiao W., Li B., Liao X. (2021). Preparation of Silk Fibroin/Hyaluronic Acid Hydrogels with Enhanced Mechanical Performance by a Combination of Physical and Enzymatic Crosslinking. J. Biomater. Sci. Polym. Ed..

[30] Friedrich R.B., Ravanello A., Cichota L.C., Rolim C.M.B., Beck R.C.R. (2009). Validation of a Simple and Rapid UV Spectrophotometric Method for Dexamethasone Assay in Tablets. Quim. Nova.

[31] Stefan N., Miroiu F.M., Socol G. (2019). Degradable Silk Fibroin—Poly (Sebacic Acid)Diacetoxy Terminated, (SF-PSADT)Polymeric Composite Coatings for Biodegradable Medical Applications Deposited by Laser Technology. Prog. Org. Coatings.

[32] Boulet-Audet M., Vollrath F., Holland C. (2015). Identification and Classification of Silks Using Infrared Spectroscopy. J. Exp. Biol..

[33] Zhang X.M., Wyeth P. (2010). Using FTIR Spectroscopy to Detect Sericin on Historic Silk. Sci. China Chem..

[34] Chen X., Knight D.P., Shao Z., Vollrath F. (2001). Regenerated Bombyx Silk Solutions Studied with Rheometry and FTIR. Polymer.

[35] Nemoto R., Nakamura S., Isobe T., Senna M. (2001). Direct Synthesis of Hydroxyapatite-Silk Fibroin Nano-Composite Sol via a Mechanochemical Route. J. Sol. Gel Sci. Technol..

[36] Landi E., Celotti G., Logroscino G., Tampieri A. (2003). Carbonated Hydroxyapatite as Bone Substitute. J. Eur. Ceram. Soc..

[37] Stipniece L., Salma-ancane K., Borodajenko N., Sokolova M. (2014). Characterization of Mg-Substituted Hydroxyapatite Synthesized by Wet Chemical Method. Ceram. Int..

[38] Weska R.F., Vieira W.C., Nogueira G.M., Beppu M.M. (2009). Effect of Freezing Methods on the Properties of Lyophilized Porous Silk Fibroin Membranes. Mater. Res..

[39] Jeong J., Kim J.H., Shim J.H., Hwang N.S., Heo C.Y. (2019). Bioactive Calcium Phosphate Materials and Applications in Bone Regeneration. Biomater. Res..

[40] Huang J., Best S.M., Brooks R.A., Rushton N., Bonfield W. (2008). In Vitro Evaluation of Nanosized Carbonate-Substituted Hydroxyapatite and Its Polyhydroxyethylmethacrylate Nanocomposite. J. Biomed. Mater. Res. Part A.

[41] Moreira Teixeira L.S., Feijen J., van Blitterswijk C.A., Dijkstra P.J., Karperien M. (2012). Enzyme-Catalyzed Crosslinkable Hydrogels: Emerging Strategies for Tissue Engineering. Biomaterials.

[42] Thakre A.A., Singh A.K. (2018). Determination of Work of Adhesion of Gelatin Hydrogels on a Glass Substrate. Mater. Res. Express.

[43] Zakharov N.A., Demina L.I., Aliev A.D., Kiselev M.R., Matveev V.V., Orlov M.A., Zakharova T.V., Kuznetsov N.T. (2017). Synthesis and Properties of Calcium Hydroxyapatite/Silk Fibroin Organomineral Composites. Inorg. Mater..

[44] Hickey T., Kreutzer D., Burgess D.J., Moussy F. (2002). Dexamethasone/PLGA Microspheres for Continuous Delivery of an Anti-Inflammatory Drug for Implantable Medical Devices. Biomaterials.

[45] Long J., Nand A.V., Bunt C., Seyfoddin A. (2019). Controlled Release of Dexamethasone from Poly(Vinyl Alcohol) Hydrogel. Pharm. Dev. Technol..

[46] Khodaverdi E., Kheirandish F., Mirzazadeh Tekie F.S., Khashyarmanesh B.Z., Hadizadeh F., Moallemzadeh Haghighi H. (2013). Preparation of a Sustained Release Drug Delivery System for Dexamethasone by a Thermosensitive, In Situ Forming Hydrogel for Use in Differentiation of Dental Pulp. ISRN Pharm..

[47] Petta D., Fussell G., Hughes L., Buechter D.D., Sprecher C.M., Alini M., Eglin D., D’Este M. (2016). Calcium Phosphate/Thermoresponsive Hyaluronan Hydrogel Composite Delivering Hydrophilic and Hydrophobic Drugs. J. Orthop. Transl..

[48] Sceglovs A., Salma-Ancane K. (2020). Novel Hydrogels and Composite Hydrogels Based on Ԑ-Polylysine, Hyaluronic Acid, and Hydroxyapatite. Key Eng. Mater..

[49] Kim M.H., Kim B.S., Lee J., Cho D., Kwon O.H., Park W.H. (2017). Silk Fibroin/Hydroxyapatite Composite Hydrogel Induced by Gamma-Ray Irradiation for Bone Tissue Engineering. Biomater. Res..

[50] Annabi N., Nichol J.W., Zhong X., Ji C., Koshy S., Khademhosseini A., Dehghani F. (2010). Controlling the Porosity and Microarchitecture of Hydrogels for Tissue Engineering. Tissue Eng. Part. B Rev..

[51] Wu W., Zhang Z., Xiong T., Zhao W., Jiang R., Chen H., Li X. (2017). Calcium Ion Coordinated Dexamethasone Supramolecular Hydrogel as Therapeutic Alternative for Control of Non-Infectious Uveitis. Acta Biomater..

[52] Aramwit P., Kanokpanont S., Nakpheng T., Srichana T. (2010). The Effect of Sericin from Various Extraction Methods on Cell Viability and Collagen Production. Int. J. Mol. Sci..

[53] Liu T., Miao J., Sheng W., Xie Y. (2010). Cytocompatibility of Regenerated Silk Fibroin Film: A Medical Biomaterial Applicable to Wound Healing. J. Zhejiang Univ. B Biomed. Biotechnol..

[54] Tremble L.F., Heffron C.C.B.B., Forde P.F. (2020). The Effect of Calcium Electroporation on Viability, Phenotype and Function of Melanoma Conditioned Macrophages. Sci. Rep..

[55] Vaz C.V., Rodrigues D.B., Socorro S., Maia C.J. (2015). Effect of Extracellular Calcium on Regucalcin Expression and Cell Viability in Neoplastic and Non-Neoplastic Human Prostate Cells. Biochim. Biophys. Acta Mol. Cell Res..

[56] Velard F., Braux J., Amedee J., Laquerriere P. (2013). Acta Biomaterialia Inflammatory Cell Response to Calcium Phosphate Biomaterial Particles: An Overview. Acta Biomater..

[57] Olkowski R., Kaszczewski P., Czechowska J. (2015). Cytocompatibility of the Selected Calcium Phosphate Based Bone Cements: Comparative Study in Human Cell Culture. J. Mater. Sci. Mater. Med..

[58] Klammert U., Reuther T., Jahn C., Kraski B., Ku A.C. (2009). Cytocompatibility of Brushite and Monetite Cell Culture Scaffolds Made by Three-Dimensional Powder Printing. Acta Biomater..

[59] Shi Z., Huang X., Cai Y., Tang R., Yang D. (2009). Size Effect of Hydroxyapatite Nanoparticles on Proliferation and Apoptosis of Osteoblast-like Cells. Acta Biomater..

